# Co-occurring symptoms of attention deficit hyperactivity disorder (ADHD) in a population-based sample of adolescents screened for depression

**DOI:** 10.1186/s12888-016-0739-3

**Published:** 2016-02-25

**Authors:** Astri J. Lundervold, Stephen P. Hinshaw, Lin Sørensen, Maj-Britt Posserud

**Affiliations:** Department of Biological and Medical Psychology, Jonas Lies vei 91, Bergen, Norway; Regional Centre for Child and Youth Mental Health and Child Welfare, Uni Research, Bergen, 5002 Norway; K. G. Jebsen Center for Research on Neuropsychiatric Disorders, University of Bergen, Bergen, 5009 Norway; Department of Psychology, University of California, Berkeley, CA USA; Department of Psychiatry, University of California, San Francisco, CA USA; Department of Child and Adolescent Psychiatry, Haukeland University Hospital, Bergen, Norway; Department of Clinical Medicine, Faculty of Medicine and Dentistry, University of Bergen, Norway

**Keywords:** Depression, ADHD, Gender difference, Adolescents

## Abstract

**Background:**

Depression is common in adolescents, with a gender bias towards girls. Symptoms associated with attention deficit hyperactivity disorder (ADHD) tend to co-occur in depressed adolescents. This may be related to common features between the two symptom domains, but co-occurring ADHD symptoms may also inflate the severity of depression. The present study investigates the frequency and influence of ADHD symptoms co-occurring with depression in a gender balanced population-based sample of Norwegian adolescents.

**Methods:**

A sample of 9614 adolescents (16–19 years) completed a questionnaire including the short version of the Mood and Feelings Questionnaire (sMFQ) and the Adult ADHD Self-Report Scale (ASRS), with items reflecting symptoms associated with depression and ADHD, respectively. The sMFQ sum score was used as a proxy for severity of depression, and adolescents with a score equal to or above the 90th percentile were defined as depressed. A high response on any of the ASRS items was used to define the presence of an ADHD symptom, and the number of high scores was used to indicate severity.

**Results:**

ADHD symptoms were frequently reported by the adolescents, with a higher frequency in girls than in boys. The gender differences were, however, minor when the analysis was restricted to the adolescents defined as depressed. Each severe symptom reported on the ASRS contributed significantly to increase the sum score on the sMFQ, and more than 20 % of the adolescents defined as depressed reported six or more symptoms within the ASRS inattention subscale.

**Conclusions:**

The results emphasize the importance of screening for symptoms associated with ADHD when assessing adolescents presenting symptoms indicating depression. Although girls reported higher frequency of symptoms within both domains, the gender bias was dependent on the overall symptom severity. Awareness of co-occurrence of symptoms and gender biases are of importance for both clinical work and future research on mental health and service use in adolescence.

**Electronic supplementary material:**

The online version of this article (doi:10.1186/s12888-016-0739-3) contains supplementary material, which is available to authorized users.

## Background

Adolescence is a period of life when problems related to emotional and behavioral function rise to unprecedented levels as part of normative development [1]. For some, these problems form the symptom clusters defining the diagnoses of depression, a disorder with a point prevalence rate reported to be as high as 11 % [2]. However, even symptoms that are below the diagnostic threshold may be harmful to their mental and somatic health (e.g., [3–6]). With more than 20 % reporting severe symptoms of depression in a population-based sample of adolescents [7], identification and treatment of adolescents in need of professional help clearly represent a serious public health concern.

Several studies have documented a high rate of co-morbidity between depression and attention deficit hyperactivity disorder (ADHD) (see e.g., [8, 9]). ADHD is a neurodevelopmental disorder with a childhood prevalence of approximately 5 % [10], and symptoms tend to persist into adulthood [11–13]. Longitudinal studies have shown that adolescents with a childhood diagnosis of ADHD tend to display symptoms of depression ([14, 15]), and persistent ADHD symptoms frequently co-occur in those adolescents. Such co-occurring symptoms of depression and ADHD are expected to increase the burden of everyday life challenges of the adolescents. First of all, the two symptom patterns may intensify each other. This has been extensively shown in studies of self-harm, where the risk increase substantially when symptoms of ADHD co-occur with depression (see e.g., [14, 15]). However, symptoms of ADHD in a depressed adolescent may be difficult to identify because symptoms of the former may not be noticed - and even such symptoms that are below the threshold for a diagnosis of ADHD may still be harmful [16–18]). When assessing an adolescent presenting symptoms of depression, a clinician should therefore be aware of symptoms that could be better described and treated if handled as part of another diagnostic category.

Secondly, co-occurring ADHD symptoms in depressed adolescents may be disregarded due to the many shared features and overlapping symptoms between the two diagnostic categories [19]. For example, restlessness and concentration difficulties are core symptoms of both diagnostic categories. When reported by an adolescent diagnosed with depression, these symptoms may thus disguise more widespread problems that are better described and treated as part of the inattention and hyperactivity-impulsivity domains of ADHD. Previous studies assessing depressive symptoms by the the Mood and Feelings Questionnaire - sMFQ [8] - have shown that these two symptoms may not exclusively appear as part of a depressive symptomatology. A study from our research group [7] showed that tiredness, restlessness and concentration difficulties showed ambiguous loadings in a bifactor model, and Sharp and colleagues [20] showed the weakest discriminative power of these symptoms in the most severe end of sMFQ. Emotional regulation is another feature that is shared: it is a characteristic of not only depression but also a core feature of many cases of ADHD [21–23]. Emotional dysregulation has even been described as a mechanism linking the two disorders [24]. Furthermore, emotional regulation is closely related to cognitive functions involving the ability to focus and shift attention and to inhibit and adapt behavior to a given situation [25], cognitive functions that are impaired in individuals with a diagnosis of depression [26–28] as well as ADHD [29–31]. However, even common features may represent different core problems, intensifying each other in the everyday life of an adolescent. Awareness about overlap between features of depression and ADHD - and also about how symptoms that are more specific to one disorder influence the other - are therefore essential when assessing adolescents presenting symptoms of depression.

The traditional gender stereotypes and differences in symptom presentation for boys and girls may further add to a clinical bias toward awareness of one symptom domain. For example, we may fail to see or acknowledge depressive symptoms in a restless boy and impulse-control symptoms in a depressed girl. It is well documented that depression is about two-fold more common in girls than in boys [7, 32], a gender discrepancy that escalates significantly during adolescence [33]. The gender bias is also substantial for ADHD, but with a male predominance. This is reflected by the boy:girl ratio of at least 3:1 commonly reported in studies of children (see e.g., [34]). A recent study presented a ratio as high as 6:1 in a population-based sample of Italian adolescents [35], wheras the gender distribution is reported to be more balanced by adulthood [36]. Less is known about the gender differences when ADHD symptoms co-occur in adolescents with depression. There are, however, indications of a more balanced gender distribution than in studies of ADHD: adult females with ADHD are more prone to depressed mood than males [37], and the high risk of suicidal attempts and ideation during adolescence in girls with ADHD [14] suggests that this pattern is true even prior to adulthood. The high co-occurrence of symptoms associated with depression and ADHD in the adolescent population thus calls for further studies of gender effects on symptoms of ADHD and depression in an adolescent community sample.

To that end, in the present study we included a gender balanced sample of 16 to 19 year old adolescents who have completed a questionnaire including symptoms of depression and ADHD. We first present the frequency of ADHD symptoms, assessed by self reports on the adult ADHD Self-report Scale (ASRS) [38], in a sample defined as depressed according to their score on the sMFQ [39]. From previous studies we expected to find a substantial co-occurrence in both boys and girls. Symptoms that are part of the *inattention* subscale of ASRS, reflecting different aspects of cognitive function, and symptoms of restlessness from the *hyperactivity-impulsivity* subscale, were expected to predominate. Furthermore, we expected that a high number of symptoms in some of those adolescents would suggest a co-morbid ADHD diagnosis. As reviewed above, co-existing ADHD symptoms in depressed adolescents may at least partly be explained by common features across the two diagnostic categories, but co-occurrence of ADHD symptoms may still intensify the level of severity of depression. We explored this issue in a second part of our investigation by a set of regression analyzes. We asked if adding information about ADHD symptoms would change a score indicating severity of depression, here defined from the total sMFQ score. Gender was included, together with its interaction with ADHD symptoms, to investigate its influence on depression severity, and we examined whether these influences are stable across different severity levels of depression.

## Methods

The present study includes data from the ung@hordaland-survey of adolescents in the county of Hordaland, Western Norway. All adolescents born between 1993 and 1995, living in the county during the spring 2012, were invited to participate. The adolescents attending high school received information via e-mail about the study, and one school hour was allocated for them to complete a questionnaire. Adolescents not in school received information by postal mail to their home addresses. The questionnaire administration was web-based and covered a broad range of mental health- and demographic background variables, including the sMFQ [39] and the ASRS [38]). The Regional Centre for Child and Youth Mental Health and Child Welfare, Uni Research Health, collaborated with Hordaland County Council to conduct the study. All adolescents gave their informed consent to participate in the study, which was approved by the Regional Committee for Medical and Health Research Ethics in Western Norway. The current study is based on the first version of quality-assured data files released in May 2012. Information about sex and year of birth were based on the personal identity number in the Norwegian National Population Register. More information about the dataset and its accessibility can be found at the website http://uni.no/en/uni-health/rkbu-vest/the-bergen-child-study/.

### Sample

Of the included adolescents (all adolescents in the county of Hordaland born between 1993 and 1995, *n*=19121), 10220 completed or filled in parts of the questionnaire, yielding a participation rate of 53 %. Almost all participants (98 %) attended a Norwegian high school at the time of the study. A total of 518 adolescents were excluded due to missing reports on some of the sMFQ items and 88 participants had incomplete ASRS data. The 9614 participants with response on all items of the two questionnaires were included. They were not different from the excluded participants on demographic variables (gender and year of birth).

### Short mood and feelings questionnaire - sMFQ

In the original version of the Mood and Feelings Questionnaire (MFQ), the respondents are asked to rate 33 items on a 3-point scale, indicating how much they have felt or acted that way during the past two weeks [39]. In the present study we included the official Norwegian version of the short version (sMFQ), with 13 items focusing on affective and cognitive symptoms (Additional file 1: Table S1). For each question the adolescents were asked to indicate how much they had felt or acted in the past two weeks. The wordings of the response categories in the Norwegian translation equal the original categories of *not true*, *sometimes true* and *true*. In the present study we defined a symptom as present when an adolescent gave a *true* response. The sum score was used as a proxy to severity of mood related problems, based on studies confirming the unidimensionality of the instrument [7, 20]. A sMFQ sum score equal to or above the 90^th^ percentile in the whole sample was used to define adolescents with a severity level suggesting a diagnosis of depression. This cutoff value was selected to match the prevalence rate of 11 % reported by Avenevoli and colleagues [2].

### Adult ADHD self-report scale - ASRS

We also included the official Norwegian translation of ASRS, of which nine items represent the subscale of *inattention* (items 1–4 and 7–11) and nine the subscale of *hyperactivity-impulsivity* (items 5–6 and 12–18) (Additional file 1: Table S2). For each of the items the participants were asked to evaluate if they had *never*, *rarely*, *sometimes*, *often*, or *very often* experienced the indicated symptom during the last six months.The responses are dichotomized in the official version, based on findings in an early study by Kessler and collaborators [38]: a symptom was evaluated as present based on a *sometimes*, *often*, or *very often* response for seven of the items (four from the *inattention* subscale), while the two latter responses indicate a symptom for the other eleven items. Although a recent study indicated the instrument’s validity for use in studies of adolescents aged 13 to 17 years [40], the validity of the Norwegian version has still not been appropriately reported upon. We therefore decided to define a symptom as present only if an adolescent used the *often* or *very often* response categories in the present study. The number of reported symptoms was used to indicate severity, and a diagnosis of ADHD was suggested if an adolescent reported six or more symptoms within the inattention or hyperactivity-impulsivity subscale.

### Statistical analyzes

The data were analyzed using R (version 3.0.1; [41]). Descriptive statistics were used to present the frequency of ASRS symptoms associated with the included sMFQ scores. To investigate the change in sMFQ score related to reports of ADHD symptoms, a set of regression analyzes were computed. The sMFQ sum score was included as the criterion variable, and the number of present ASRS symptoms was included as predictor together with gender and the interaction between gender and the ASRS symptoms. Significant results were followed by hierarchical analyzes to investigate the contribution of gender when the ADHD symptoms were controlled. Effect sizes of significant mean differences were calculated and interpreted according to Cohen [42], in which a *d* value of.20 = small,.50 = medium, and.80 = large.

## Results

### Demographics and symptom reports

Among the 9614 adolescents who participated in the study, 53.5 % were girls. The gender distribution was not different between the three birth-cohorts - i.e., born in 1993 (55.5 %), 1994 (52.7 %), or 1995 (53.1 %). Mean self-reported age was 16.95 (SD =.86) (16.97 (.86) in girls and 16.93 (.87) in boys). The majority (98 %) of the adolescents were high school students (98.3 % in girls and 97.6 % in boys), and the proportion of immigrants, defined as having both parents born outside Norway, was 5.6 % (5.7 % in girls and 5.5 % in boys). The gender differences were statistically non-significant for all these demographic variables.

As known from a previous study [7], girls obtained a higher mean sum score on the sMFQ (7.4 (6.1)) than boys (4.2 (4.9)), *t* (1,9568.4) =28.7,*p*<.001,*d*=.58). Albeit with lower effect-sizes, the mean sum scores for girls (15.9 (6.0)) were also significantly higher than for boys (14.0 (6.4)) on the ASRS *inattentiont* (1,9253.5)=15.2,*p*<.001,*d*=.31) and the *hyperactivity-impulsivity* subscales (12.6 (5.3) and 11.1 (5.7)), *t* (1,9187.2)= 13.4,*p*<.001, *d*=.28). Table 1 shows the frequency of adolescents within each gender group showing symptoms of depression and ADHD according to their responses on the sMFQ and ASRS items, respectively. The frequency of girls was higher than for boys on all 13 sMFQ items, on seven of the nine items from the ASRS *inattention* subscale, and five of nine from the *hyperactivity-impulsivity* subscale. Within the ASRS subscales, the highest frequencies were found on items reflecting motor uneasiness (#5) and feeling of restlessness (#13), and cognitive function reflecting careless mistakes when working on a boring or difficult project (#7), and problems to initiate (#4) and stay focused on a task (#11).

**Table 1:** Frequency (%) of adolescents reporting symptoms according to sMFQ and ASRS

	sMFQ			ASRS		
	Girls	Boys	*p*-value	Girls	Boys	*p*-value
Item 1	20.5	7.0	**	22.6	15.7	**
Item 2	12.1	6.2	**	13.0	9.7	**
Item 3	26.0	12.2	**	9.8	9.5	ns
Item 4	14.2	10.2	**	34.5	23.9	**
Item 5	14.0	5.8	**	51.3	43.1	**
Item 6	11.0	2.0	**	12.6	12.6	ns
Item 7	19.9	9.3	**	26.5	24.9	ns
Item 8	8.3	3.2	**	43.5	33.9	**
Item 9	8.4	3.6	**	12.5	8.2	**
Item 10	15.1	7.1	**	18.7	11.5	**
Item 11	7.3	3.7	**	25.8	16.0	**
Item 12	10.5	4.7	**	2.5	2.4	ns
Item 13	7.6	3.6	**	23.9	21.2	*
Item 14				15.6	9.4	**
Item 15				14.7	9.0	**
Item 16				7.5	5.6	**
Item 17				4.8	4.9	ns
Item 18				4.4	3.6	ns

** *p*<.001, *p value <.05: comparisons between the two genders within each scale

ASRS I = ASRS *inattention* subscale; ASRS H = ASRS *hyperactivity-impulsivity*

### Percentage of depressed adolescents reporting co-occurring ADHD symptoms

Table 2 shows the frequency of depressed and non-depressed adolescents reporting symptoms of ADHD within the *inattention* and the *hyperactivity-impulsivity* subscales. The percentages of ADHD symptoms were calculated separately for girls and boys. Overall, a substantial proportion of the adolescents reported ADHD symptoms, with a higher frequency for the symptoms within the *inattention* than the *hyperactivity-impulsivity* subscale. However, the highest percentages were found for one item from the latter subscale: more than 70 % of the girls and 65 % of the boys reported severe motor uneasiness when they had to sit down for a long time (#5). In addition, more than 45 % reported that they feel restless or fidgety (#13). It should also be noted that the first of these two symptoms of restlessness was also reported by a high number of adolescents defined as not depressed. Girls in the not-depressed group reported higher scores than the boys on most ASRS items, while gender differences were statistically significant for only three ADHD symptoms within the depressed subgroup: the frequency of girls reporting problems related to initiation of a task (#4) was higher than for boys, while the frequency was higher for boys on items related to waiting for turn taking (#17) and the tendency to interrupt others when they are busy (#18).

**Table 2:** Frequency (%) among the girls and the boys reporting ADHD symptoms when defined as depressed and non-depressed

ASRS I items	1	2	3	4	7	8	9	10	11
Depressed									
Girls (*n*=856)	43.6	31.7	22.2	57.5**	45.7	64.6	30.7	30.6	45.0
Boys (*n*=242)	38.8	31.4	24.8	48.3	49.6	58.3	.25.6	30.2	40.9
Not depressed									
Girls (*n*=4292)	18.4**	9.2	7.4*	29.4**	22.7	39.3**	8.9*	16.4**	22.0**
Boys (*n*=4224)	14.3	8.5	8.7	22.5	23.5	32.6	7.2	10.4	14.6
ASRS H items	5	6	12	13	14	15	16	17	18
Depressed									
Girls	70.7	24.2	7.7	45.2	31.9	23.8	11.7	10.5	9.6
Boys	64.5	30.2	.10.7	47.8	30.2	23.6	.16.5	18.6**	14.9*
Not depressed									
Girls	47.4**	10.3*	1.6	19.6	12.3**	12.9**	6.7*	3.7	3.4
Boys	41.9	11.6	2.0	19.7	8.2	8.2	4.9	4.2	3.0

ASRS I = ASRS *inattention* subscale; ASRS H = ASRS *hyperactivity-impulsivity* subscale

Depression: sMFQ ≥14; Not depressed: sMFQ <14. *p value <.05; **p value <.001

Table 3 suggests that a fairly high number of depressed adolescents showed a cluster of symptoms that may qualify for a diagnosis of ADHD. More than 20 % reported at least six symptoms within the *inattention* subscale. The percentage was lower within the *hyperactivity-impulsivity* subscale, but still included about 10 %. The gender distribution was biased towards girls when using a common cutoff score for depression (sMFQ ≥14). The analyzes were therefore reconducted using the 90^th^ percentile within each gender group. The bottom rows of Table 3 show that this procedure left the results almost unchanged.

**Table 3:** Frequency among the girls and the boys with a given number of ADHD symptoms (n) co-occurring with depression

ADHD symptoms (n)	*<4*		4		5		*>5*	
	Girls	Boys	Girls	Boys	Girls	Boys	Girls	Boys
Common cutoff for girls & boys								
ASRS I	49.9	51.7	11.4	14.5	12.5	9.1	26.2	24.8
ASRS H	61.9	58.3	16.7	17.8	11.4	8.7	9.9	15.3
Gender specific cutoffs								
ASRS I	46.9	60.0	10.2	11.8	12.3	7.9	30.6	20.3
ASRS H	59.0	64.9	17.6	16.0	12.1	7.5	11.3	11.6

ASRS I = ASRS *inattention* subscale; ASRS H = ASRS *hyperactivity-impulsivity* subscale;

Cutoff for boys and girls: sMFQ ≥ 14 (856 girls and 242 boys defined as depressed)

Gender specific cutoffs: sMFQ ≥17 for girls (529 girls) and sMFQ ≥11 for boys (507 boys)

### Co-occurring ADHD symptoms and severity of depressive symptoms

The influence of co-occurring reports of ADHD symptoms on severity of depressive symptoms was investigated by a regression analysis, with sMFQ sum score serving as the criterion variable, and the number of ADHD symptoms as a predictor along with gender and the interaction between the two. The results were statistically significant for both ASRS subscales (*p*<.001), explaining 25 % and 20 % of the variance of the sMFQ sum score, respectively (Table 4). The total sMFQ score was more strongly explained by the presence of ADHD symptoms in girls than in boys. This was confirmed by the hierarchical analysis, showing a significant added value of gender (*p*<.001) in both models when number of ADHD symptoms was analyzed (*inattentionF*(1,9611) =635.7; *hyperactivity-impulsivityF*(1,9611) =621.8). According to the estimated regression coefficients, the total sMFQ score in girls changed by between 1 and 2 units, with a somewhat higher change-score for the *hyperactivity-impulsivity* than the *inattention* subscale. A stronger change score for the *hyperactivity-impulsivity* subscale was also found in boys. There were also statistically significant interactions between gender and ADHD symptoms defined both within the *inattention*, *F*(1,9610) = 30.6, and the *hyperactivity-impulsivity*, *F*(1,9610) = 47.2, subscales, meaning that the effect of gender was more similar, in terms of predictions from the ADHD symptoms, in boys and girls at the highest than the lowest end of the sMFQ sum score.

**Table 4:** The explained variance in the total sMFQ score from number of severe ASRS problems and gender

*sMFQ sum*	F-test	*df*	*p*-value	*R* ^2^	I:B	Change:F	Change:B
ASRS Inattention	1067.0	3/9610	<.001	.25	–2.13**	1.26 **	.98**
ASRS HypImp	800.1	3/9610	<.001	.20	–2.00*	1.62**	1.22**

Estimated regression coefficients: I:B=intercept difference: boys minus girls; Change:F=change in score on the dependent variable with girls as a reference; Change:B= change in score for boys

*p value <.05; **p value <.001

## Discussion

We investigated the frequency of co-occurring ADHD symptoms in adolescents defined as depressed, and asked if these symptoms contributed to inflate the severity level of depression. The results revealed a high frequency of co-occurring symptoms in depressed adolescents. A very high number of the adolescents reported ADHD symptoms included in the *inattention* subscale of an ADHD diagnosis, with more than 20 % reporting as many as six or more symptoms within this scale. In addition, about two thirds of the depressed subgroup reported symptoms reflecting restlessness. The frequency of ADHD symptoms was much lower in adolescents defined as not depressed. However, it should be noted that restlessness was reported by more than 40 % even in this latter, not depressed subgroup. ADHD symptoms contributed significantly to the score used as a proxy for severity of depression, with a one unit change on the depression scale for each added ADHD symptom. Overall, girls reported symptoms of depression and ADHD more frequently than boys, but the gender differences were minor when analyzed within the subgroup of adolescents defined as depressed. The score representing the severity of depressive symptoms was more strongly influenced by ADHD symptoms in girls than in boys, with a stronger effect of symptoms within the *hyperactivity-impulsivity* than the *inattention* subscale for both genders. However, the gender difference was lower at the higher than lower end of the depression severity level.

A high rate of comorbidity between psychiatric disorders is extensively documented (see e.g., [8, 32, 43]). The contribution of the present study was to show the high frequency of ADHD symptoms in a gender-balanced population-based sample of adolescents screened for depression. Based on the findings from this study, both clinicians and researchers should be attentive to co-existing ADHD symptoms whenever an adolescent reports a high number of symptoms of depression on a scale like sMFQ. Depression was selected as the outcome variable in the present study, but the converse may very well also be true. A more general take-home message is thus that youth themselves more often than not experience problems within the two domains simultaneously; restricting assessment and treatment to only one of the diagnostic categories may be misleading.

Restlessness was reported by a substantial part of the entire adolescent sample in the present study, emphasizing that restlessness and associated problems represent a substantial challenge in the population of adolescents at large. A peak was, however, found in the depressed subsample, where restlessness was reported by around two third of the adolescents. It should be noted that restlessness associated with depression has been described as part of a cluster of somatic symptoms, including fatigue, anhedonia, and concentration difficulties (see [44]). The present findings suggest that anhedonia represents a symptom that is specific to depression, while the shared value of concentration difficulties was shown by the widespread association with the ADHD symptoms included in the *inattention* subscale. We thus confirmed that restlessness and cognitive function represent two of the main co-occurring ADHD symptoms in depressed adolescents. At a more general level, it also supports previous psychometric studies showing that these symptoms may appear both in conjunction with and independent of depressive symptomatology [7, 20]. Independence, suggesting that the symptoms within the two symptom domains may not capture exactly the same core problem, was indicated by the change score in the regression analysis. A much lower change would have been expected with a more perfect feature overlap across the two symptom domains. Further psychometric studies are needed to investigate this issue in more detail.

The inattention items included in the ASRS cover a much wider range of cognitive dysfunction than the concentration item from the sMFQ. The widespread association between the latter and almost the full range of items in the *inattention* subscale confirms the importance of cognitive dysfunction in both disorders. This finding is well documented in studies of children [45–47] and adults [48–50] with ADHD, and the importance of cognitive function for remediation of depression has been increasingly recognized during the last few years (see e.g., [51]. Cognitive function is obviously essential for adolescents attending high school, and cognitive dysfunction is among the strongest predictive factors for everyday functioning in adult life [1, 52]. Identification of cognitive dysfunction that is not directly part of the diagnostic criteria for depression may thus be essential to the outcome when treating adolescents with a full or subthreshold diagnosis of depression. This is well exemplified by studies of suicidal behavior of adolescents, showing the importance of ADHD symptoms [14, 15, 53] and symptoms of depression [3–6]. Cognitive deficits defined as problems related to response-inhibition [54] and emotional regulation [55] have been described as core risk factors for self-harm behavior in girls with ADHD. Furthermore, Nock et al. [56] have shown that more than 50 % of those with a recent history of self-injury met the criteria for both an externalizing and internalizing disorder. For instance, the combination of being impulsive and highly depressed may increase the risk of a depressed adolescent in a moment of despair to engage in self-harm or suicidal behavior. The importance of such overlap, at least in females with a history of ADHD, [14, 43, 57], should not be underestimated. The present results also emphasize awareness from teachers and others who observe adolescents at school. Between 40 and 50 % of the participants reported that they fidget or squirm with their hands or feet when they had to sit down for a long time, and a high proportion did also report difficulty keeping attention when they were doing boring or repetitive work. Teachers can easily detect this kind of behavior, and the present study suggests that they are related to and probably for some adolescents disguise problems better defined as part of depression.

The higher depression-related scores in girls than in boys confirmed results from several previous studies [7, 32, 58, 59]. The predominance of girls reporting ADHD symptoms was more surprising given the male predominance reported in previous studies of children [34] and adolescents [35]. A gender bias in self-reports may explain the high severity level reported across almost all items of the sMFQ and the ASRS scales. This conclusion is supported by studies showing that females tend to be more ready to acknowledge and disclose discomfort than males [60, 61]. Furthermore, this tendency may have been aggravated by the high frequency of girls showing depressive symptoms, because depression is known to make people more critical of their overall function [62, 63]. Response-style can, however, not fully explain the results in the present study. Both girls and boys reported a high number of ADHD symptoms, along with a score used to define depression, and the change score of ADHD symptoms on the full sMFQ score was more gender balanced in the higher and most severe than the lower end of the scale. The overall higher symptom scores reported by girls may rather illustrate the importance of choice of informant. A high boy:girl ratio is primarily shown in studies using teachers and/or parents as informants, while self-reports gives an almost equal gender distribution in studies of adults with ADHD [36, 64]. The girls reporting ADHD related symptoms in the present study may thus have been disregarded or misinterpreted if they had been evaluated by other informants [65]. Further studies are clearly needed to ascertain the source effect of gender biases in how individuals present their mental health at different ages. Future studies should also include search for etiological factors of symptom patterns in more general, based on the intriguing results in a recent paper from Moffitt et al. [66], showing that adults commonly show symptoms indicating an ADHD diagnosis even without childhood onset. From the results of the present study, misclassifications of behavioral and emotional problems in adolescents are clearly expected if the assessment is restricted to the face value of symptoms described within a given diagnostic category.

### Strengths and limitations

The gender-balanced, population-based sample of adolescents was a main strength of the present study, together with the stringent procedure of completion of a standardized questionnaire administrated at school for most of the participants. However, the study has several limitations. One limitation is related to the source effect mentioned above. However, reports from adolescents are likely to be the main input of information from adolescents, making the results relevant for what a clinician should expect when assessing an adolescent. The use of screening instruments rather than a stringent diagnostic procedure to assess depression and ADHD diagnosis is another limitation of the present study. The sMFQ sum score was used both to define depression and as an outcome variable when analyzing severity of symptoms based on the unidimensionality of the instrument shown in previous psychometric studies [7, 20]. We could have used a similar procedure for the ADHD symptoms, in that several studies have documented a general factor across different instruments assessing ADHD in previous studies [45–50]. A psychometric study of adolescents completing the Norwegian translation of ASRS is, however, still lacking, and we therefore focused on ADHD symptoms as reported for single items rather than sum scores. This was also considered to be appropriate, as our present focus was on ADHD-related symptoms rather than on the diagnostic category of ADHD. Finally, the results demonstrate associations; information about casual relationships awaits longitudinal studies.

#### Clinical implications and future directions

Being a vulnerable age, adolescence represents an extremely important window for interventions to reduce the burden of mental disorders across the lifespan. A majority of mental disorders seen in adults have their onset in adolescence. The findings of the present study emphasize the importance of a broad perspective on mental health. Introduction of screening instruments covering overlapping aspects of mental health may lead to better and earlier detection of the adolescents in need of help. Routine screening in the primary care, followed by diagnostic assessment, has been described as an effective strategy for selecting cases in need of help [67]. Given the high frequency of symptoms, further studies should investigate the impact of preventive treatment programs in this age group [68]. In that adolescents are vulnerable to problems related to lack of cognitive and emotional control, management of situations with a high load on these functions should be among the core features of such programs. Further studies should include information enabling investigation of moderating factors such as peer relation problems [69, 70], sleep [71] and socio-economic status [72], and investigate the long-term effects of symptoms that presently are associated with the diagnoses of depression and ADHD.

Distinguishing between symptoms reflecting problems that are part of normal development and those that should elicit enough worry to engage professional help, and between diagnostic categories sharing common features, are both common challenges when evaluating clinical caseness. The participants may not have had the same interpretation of the items reflecting concentration difficulties/inattention and restlessness in the two scales. Although diagnostic manuals like DSM-5 apply specific characteristics to a given diagnosis, symptoms may be reported for different reasons. Furthermore, symptom reports may even be gender specific and specific to a given period of life. Further longitudinal studies, as the studies conducted by the research group at UC Berkeley focusing on girls [14] and the Dunedin study in New Zealand [66], are therefore essential to improve our knowledge about symptoms associated with mental health problems through the lifespan.

## Conclusions

The present large-scale study of adolescents showed a strong overlap between symptoms associated with depression and symptoms associated with ADHD. Knowing that adolescence is an extremely vulnerable period for mental health problems, broad perspectives on assessment and intervention programs are called for. Girls reported a higher number of severe problems than boys within both problem domains, with a strong influence from each severe ADHD-related problem on their reports of depressive symptoms. A more gender-balanced report was found among adolescents with a score suggesting severe depression. If left undetected, these problems are expected to lead to serious present and future problems. The present findings suggest that screening instruments covering overlapping aspects of symptoms reflecting emotional and behavioral problems are necessary to obtain a better and earlier detection of the adolescents in need of help.

## Additional file

Additional file 1
**Supplementary Tables.** (PDF 12 kb)
