# Supplementary material for: Co-occurring symptoms of attention deficit hyperactivity disorder (ADHD) in a population-based sample of adolescents screened for depression
Source: BMC Psychiatry. 2016 Feb 25;16:46. doi: 10.1186/s12888-016-0739-3 (PMC4768418; doi:10.1186/s12888-016-0739-3)
Supplement: Additional file 1 — Supplementary Tables. (PDF 12 kb) [file 12888_2016_739_MOESM1_ESM.pdf]

Supplementary Tables

Table S-1- The items of the sMFQ

**Item 1**	I felt miserable or unhappy
**Item 2**	I didn't enjoy anything
**Item 3**	I felt so tired I just sat around and did nothing
**Item 4**	I was restless
**Item 5**	I felt I was no good any more
**Item 6**	I cried a lot
**Item 7**	I found it hard to think properly or concentrate
**Item 8**	I hated myself
**Item 9**	I was a bad person
**Item 10**	I felt lonely
**Item 11**	I thought nobody really loved me
**Item 12**	I though I could never be as good as other kids
**Item 13**	I did everything wrong

Table S-2: Items included in the ASRS

**Item 1**	Trouble wrapping up the final details of a project, once the challenging parts have been done?
**Item 2**	Difficulty getting things in order when you have to do a task that requires organization?
**Item 3**	Problems remembering appointments or obligations?
**Item 4**	When you have a task that requires a lot of thought, how often do you avoid or delay getting started?
**Item 5**	Fidget or squirm with your hands or feet when you have to sit down for a long time?
**Item 6**	Feel overly active and compelled to do things, like you were driven by a motor?
**Item 7**	Make careless mistakes when you have to work on a boring or difficult project?
**Item 8**	Difficulty keeping your attention when you are doing boring or repetitive work?
**Item 9**	Difficulty concentrating on what people say to you, even when they are speaking to you directly?
**Item 10**	Misplace or have difficulty finding things at home or at work?
**Item 11**	Distracted by activity or noise around you?
**Item 12**	Leave your seat in meetings or other situations in which you are expected to remain seated?
**Item 13**	Feel restless or fidgety?
**Item 14**	Difficulty unwinding and relaxing when you have time to yourself?
**Item 15**	Find yourself talking too much when you are in social situations?
**Item 16**	When in a conversation, find yourself finishing the sentences before they can finish them themselves?
**Item 17**	Difficulty waiting your turn in situations when turn taking is required?
**Item 18**	Interrupt others when they are busy?
